# Clustering of Dietary Patterns, Lifestyles, and Overweight among Spanish Children and Adolescents in the ANIBES Study

**DOI:** 10.3390/nu8010011

**Published:** 2015-12-28

**Authors:** Carmen Pérez-Rodrigo, Ángel Gil, Marcela González-Gross, Rosa M. Ortega, Lluis Serra-Majem, Gregorio Varela-Moreiras, Javier Aranceta-Bartrina

**Affiliations:** 1 FIDEC Foundation, University of the Basque Country, Gurtubay s/n, Bilbao 48010, Spain; carmenperezrodrigo@gmail.com; 2 Department of Biochemistry and Molecular Biology II, Institute of Nutrition and Food Sciences, Centre of Biomedical Research, University of Granada, Campus de la Salud, Avda. del Conocimiento, Armilla, Granada 18100, Spain; agil@ugr.es; 3 ImFINE Research Group, Department of Health and Human Performance, Technical University of Madrid, C/Martín Fierro 7, Madrid 28040, Spain; marcela.gonzalez.gross@upm.es; 4 Department of Nutrition, Faculty of Pharmacy, Complutense University of Madrid, Plaza Ramón y Cajal s/n, Madrid 28040, Spain; rortega@ucm.es; 5 Research Institute of Biomedical and Health Sciences, Universidad de Las Palmas de Gran Canaria, Facultad de Ciencias de la Salud, C/Doctor Pasteur s/n, Trasera del Hospital, Las Palmas de Gran Canaria 35016, Spain; lluis.serra@ulpgc.es; 6 Department of Pharmaceutical and Health Sciences, Faculty of Pharmacy, CEU San Pablo University, Urb. Montepríncipe, Crta. Boadilla Km. 5.3, Boadilla del Monte, Madrid 28668, Spain; gvarela@ceu.es or gvarela@fen.org.es; 7 Spanish Nutrition Foundation (FEN), C/General Álvarez de Castro 20. 1a pta, Madrid 28010, Spain; 8 Department of Preventive Medicine and Public Health, University of Navarra, C/Irunlarrea 1, Pamplona 31008, Spain

**Keywords:** cluster analysis, dietary patterns, physical activity, sedentary behavior, overweight, children, adolescents

## Abstract

Weight gain has been associated with behaviors related to diet, sedentary lifestyle, and physical activity. We investigated dietary patterns and possible meaningful clustering of physical activity, sedentary behavior, and sleep time in Spanish children and adolescents and whether the identified clusters could be associated with overweight. Analysis was based on a subsample (*n* = 415) of the cross-sectional ANIBES study in Spain. We performed exploratory factor analysis and subsequent cluster analysis of dietary patterns, physical activity, sedentary behaviors, and sleep time. Logistic regression analysis was used to explore the association between the cluster solutions and overweight. Factor analysis identified four dietary patterns, one reflecting a profile closer to the traditional Mediterranean diet. Dietary patterns, physical activity behaviors, sedentary behaviors and sleep time on weekdays in Spanish children and adolescents clustered into two different groups. A *low physical activity-poorer diet* lifestyle pattern, which included a higher proportion of girls, and a *high physical activity, low sedentary behavior, longer sleep duration, healthier diet* lifestyle pattern. Although increased risk of being overweight was not significant, the Prevalence Ratios (PRs) for the *low physical activity-poorer diet* lifestyle pattern were >1 in children and in adolescents. The healthier lifestyle pattern included lower proportions of children and adolescents from low socioeconomic status backgrounds.

## 1. Introduction

The prevalence of overweight and obesity has been steadily increasing worldwide over the past decades [1]. Obesity in children is of particular concern because of its rapid rate of increase and the potential negative impact on health and well-being during childhood and beyond.

Childhood obesity rates in Spain are amongst the highest in OECD (Organization for Economic Co-operation and Development) countries [2]. Despite high-quality data that support an overall leveling off of this epidemic among children and adolescents in Australia, Europe, Japan, and the United States, there is evidence for heterogeneity in obesity trends across socioeconomic groups, suggesting less evident leveling off in groups with lower socioeconomic status (SES) [2].

Overweight and obesity result from an imbalance between energy intake and energy expenditure, which leads to weight gain. Identifying important behaviors related to energy balance and their determinants within a specific target group is a key step to design effective obesity prevention interventions [3]. Weight gain has been associated with various specific behaviors related to diet, sedentary lifestyle, and physical activity [4,5]. More recently, sleeping habits have been reported to be possibly relevant for energy balance [6].

Whereas most research has focused on specific nutrient and food intake, overall dietary patterns (DPs) have drawn attention in the past decade because DPs consider all food and nutrient intakes and may account for the cumulative and interactive effects of foods and nutrients [3,7]. Dietary patterns have been used as exposures for many health outcomes [3,8], including obesity [9,10,11,12,13,14]. A number of studies have investigated DPs in children and adolescents. Several similar DPs have been described across these studies, such as a pattern that includes higher consumption of fruit, vegetables, and fish. A DP combining higher intakes of snacks and other energy-dense foods has also been described in several studies [15,16]. DPs among Spanish children and adolescents were analyzed in the enKid study (Feeding Habits and Nutritional Status in Spanish Children and Youth) in 1998–2000 [17].

No single element can be identified as a universal causal factor in the current obesity epidemic; many distinct behaviors and determinants at different levels influence a more positive energy balance [3]. Many of these behaviors are interrelated and may result in combined effects on health. Clustering, or the co-existence of groups of people who share similar characteristics, is a concept that has been successfully applied to understanding the relationships between different lifestyle behaviors [9,10]. The rationale underlying this approach acknowledges that there are multivariate and interactive influences on lifestyles [18,19].

Exploratory data-driven methods, such as cluster analysis or latent class analysis to investigate lifestyle patterns, have become increasingly common. In recent years a number of studies have used these methods to gain insight and better understand the relationships between diet, physical activity, and sedentary behavior among children and adolescents, as well as the possible cumulative effect of an unhealthy clustering of these behaviors on the development of overweight and obesity [20,21,22,23]. However, controversy exists surrounding the co-occurrence as well as their association with children and adolescent overweight [10,18].

To date, limited information is available on health-related behavior patterns among Spanish children and adolescents. Interventions that are appropriately targeted and that effectively consider multiple behavioral changes may be more cost effective and gain adherence from the most in need individuals or groups [24].

The aims of the study were (a) to identify dietary patterns among Spanish children and adolescents; (b) to investigate whether energy balance-related behaviors cluster into meaningful patterns in Spanish children and adolescents; (c) to describe sociodemographic correlates of the identified lifestyle patterns; and (d) to study the association of these correlates with overweight.

## 2. Methods

Data were obtained from the ANIBES study. ANIBES is an observational cross-sectional survey conducted in a random multistage sample of the Spanish population aged 9–75 years, living in municipalities of at least 2000 inhabitants. The aim of the survey was to evaluate energy intake and energy expenditure in a nationally representative sample of the population in Spain.

Sampling procedures and methods have been described elsewhere in detail [25,26]. Briefly, the sample for the ANIBES Study was designed based on 2012 census data published by the INE (*Instituto Nacional de Estadística*/Spanish Bureau of Statistics) for gender, age, habitat size and region. A multistage stratified sampling procedure was used, with random selection of households within municipalities and age and gender quotas for individuals within households. Interlocked quotas were established for age within region and habitat size within region. The sample selection procedure was based on random routes. In order to ensure the representativeness of the sample, 128 sampling points were used.

The final study sample consisted of 2009 individuals (1013 males, 50.4%; 996 females, 49.6%). In addition, a boost sample was recruited for the youngest age groups (9–12 years; 13–17 years, and 18–24 years) so as to include at least 200 individuals per age group. For this analysis, the final sample plus boost consisted of 213 children aged 9–12 years and 211 adolescents aged 13–17 years. Data were collected between mid-September 2013 and mid-November 2013.

The final protocol was approved by the Ethical Committee for Clinical Research of the Region of Madrid, Spain. Informed parental and student consent was required for each component of the study.

### 2.1. Measurements

#### 2.1.1. Lifestyle Factors

##### Diet

Dietary intake was assessed by means of a face-to-face 24-h recall of the one-day intake, as well as with a three-day record kept by means of a tablet device (Samsung Galaxy Tab 2 7.0) on 2 consecutive weekdays and 1 weekend day, which included all foods and beverages consumed at home and away from home. Children were assisted by their parents or guardians to complete the food records and face-to-face interview.

Food record inputs were received in real time, then checked and coded by trained coders who were supervised by dieticians. Food, beverages, and energy and nutrient intakes were calculated using software (VD-FEN 2.1) that was newly developed for the ANIBES study by the Spanish Nutrition Foundation and is based mainly on expanded and updated Spanish food composition tables [27]. A food picture atlas was used to assist in assigning weights to portion sizes of foods consumed. Food and beverage consumption data were grouped into 16 food groups, 45 subgroups, and 754 food items, for in-depth analysis, based on the structure of the food composition database according to similarities in nutrient profile (supplementary materials Table S1). The input variables for dietary pattern analysis were the average weight consumed (g/day) by each individual (three-day food record plus one-day 24-h recall) from 38 food groups. Food groups were used to further collapse dietary intake data in order to avoid missing data from non-consumers of episodically consumed foods. Z-scores for each food group were calculated.

##### Physical Activity

Physical activity data were collected by face-to-face interview using the validated International Physical Activity Questionnaire (IPAQ) for children and adolescents, modified and validated according to the HELENA (Healthy Lifestyle in Europe by Nutrition in Adolescence) study for children and adolescents [28]. Additionally, objective measurements of physical activity were obtained in a subsample of 167 adults and 39 children, using an ActiGraph accelerometer (models GT3x and GT3x+; ActiGraph, Pensacola, FL, USA) during 3 full consecutive days. The validation of the modified version in HELENA study [28] found significant, but modest correlations (±0.20) comparing the modified IPAQ results with accelerometer data, and a higher validity in the older adolescents in comparison with the younger ones. Preliminary analyses of ANIBES accelerometer data are in line with this, showing modest significant correlations with vigorous physical activity (*r* = 0.26).

Total minutes per week were computed for moderate to vigorous physical activity based on the IPAQ guidelines for data processing and analyses [29]. Data were cleaned and truncated based on IPAQ guidelines and previous research [30]. Additionally, the total minutes per week of commuting-related physical activity (walking, biking) were computed. IPAQ data was used for this analysis. Z-scores of minutes per day for each type of activity were calculated.

##### Sedentary Behaviors

Sedentary behaviors were assessed using the questionnaire validated in the HELENA study [28]. This questionnaire included daily minutes of the following sedentary activities: television viewing, playing computer games, playing video console games, non-school-related Internet use, school-related Internet use, and studying or homework (not including classroom time). The average time spent per day engaged in these sedentary activities was calculated. Screen time (*i.e.*, time spent in front of a screen, such as that of a computer, tablet, smartphone, or console game) was assessed separately for weekdays and weekend days. Mean television, computer, and total screen time per day were calculated. For the analyses, total minutes per day (min/d) of screen time were considered. Weighted mean duration of each behavior per day ((5 × weekday min/d + 2 × weekend min/d)/7) was derived and summed to provide the measure of screen time used in this analysis.

##### Sleep Duration

Sleep habits included the number of hours each child or adolescent slept per night, on average, and were reported separately for weekdays and weekend days. In this analysis, only weekdays (h/d of sleep duration) were considered because sleep on weekdays is more likely to be regular and thus more representative of usual sleep duration [31,32].

#### 2.1.2. Body Measurements

Anthropometric measurements were taken individually by trained interviewers, following international standard procedures previously tested in two pilot studies [33], as follows. Height was assessed in triplicate using a stadiometer (model 206; Seca, Hamburg, Germany) and recorded to the nearest 0.1 cm. Weight was assessed while wearing light clothing or underwear, using a Seca 804 weighing scale, and recorded to the nearest 0.1 kg. Waist circumference was assessed in triplicate using a Seca 201 tape measure and recorded to the nearest 0.1 cm. Body mass index (BMI) was calculated as body weight in kilograms divided by the square of body height in meters. Overweight status (overweight, obese) was calculated using age- and sex-specific cutoff values according to the criteria of Cole *et al.* [34], which have been adopted by the International Obesity Task Force.

#### 2.1.3. Covariates

##### Parental Education

The education levels were established in accordance with the Spanish educational system. After preliminary analysis of the distribution of the variable, categories were collapsed and recoded into a 3-point scale, as follows: (1) low (less than 7 years of education; primary school or less); (2) medium (7–12 years of education; lower to higher secondary education); and (3) high (13 years or more of education; higher vocational, college and university studies).

##### Socioeconomic Status (SES)

Socioeconomic status was classified based on parental education (six categories) and occupation (12 categories) according to National Association of Opinion and Market Research (ANEIMO) criteria, which adapt to the Spanish context the World Association for Market, Social and Opinion Research (ESOMAR) criteria. This information is then classified into low, mid-low, mid-mid, mid-high, and high socioeconomic class. After preliminary analysis of the distribution of the variable for this analysis, the categories were collapsed and recoded into a 3-point scale, as follows: (1) low; (2) mid-low; and (3) high (mid-mid, mid-high and high) SES levels.

### 2.2. Data Cleaning

Detailed data cleaning procedures have been previously described [25,26]. Participants were considered fully eligible if verified that their three-day food records had been adequately recorded using the tablet. Provided that participants had fulfilled previous data cleaning stages, they remained in the database if they had successfully completed both face-to-face interviews during fieldwork and had measured weight, height, and waist circumference data. Of the initial sample of 486 children and adolescents recruited, 62 individuals were excluded; 424 individuals (213 children aged 9–12 years and 211 adolescents aged 13–17 years) satisfied the inclusion criteria. Outliers (±3 SD) for energy intake were excluded in this analysis.

### 2.3. Data Analysis

All statistical tests were performed using IBM SPSS Statistics for Windows, Version 22.0 (IBM Corp., Armonk, NY, USA). Descriptive statistics were computed for each variable.

#### 2.3.1. Dietary Patterns

Exploratory factor analysis was performed to identify underlying dietary patterns, using the average weight consumed (g/d) by each individual from 38 food groups as input variables. Bartlett’s test of sphericity and the Kaiser-Meyer-Olkin (KMO) measure of sampling adequacy were used to verify the appropriateness of factor analysis. To assess the degree of intercorrelations between variables, we adopted a value >0.60 for the KMO. Factors were also orthogonally rotated (the varimax option) to enhance the difference between loadings, which allowed easier interpretability. Factors were retained based on the following criteria: factor eigenvalue >1.4, identification of a break point in the scree plot, the proportion of variance explained, and factor interpretability [35]. The strength and direction of the associations between patterns and food groups were described through a rotated factor loading matrix. Food groups with factor loadings >0.30 and communality >0.20 were retained in the patterns identified. The factor score for each pattern was constructed by summing observed intakes of the component food items weighted by the factor loading. A high factor score for a given pattern indicated high intake of the foods constituting that food factor, and a low score indicated low intake of those foods.

#### 2.3.2. Lifestyle Patterns

To identify clusters with similar dietary patterns, physical activities, sedentary activities, and sleeping habits, a combination of hierarchical and non-hierarchical clustering analysis was used [36]. The variables used had different arithmetic scales; thus, Z-scores were calculated to standardize the data set before clustering, to avoid a greater contribution to the distance of variables having larger ranges than variables with smaller ranges. Univariate and multivariate outliers (>3 SD) were removed. First, hierarchical cluster analysis was performed using Ward’s method, based on squared Euclidian distances. Several possible cluster solutions were identified and compared to inform the next step, considering the coefficients and fusion level. A non-hierarchical k-means clustering procedure was used, specifying the number of clusters identified in the first step, using a random initial seed and 10 iterations in order to further refine the preliminary solution by optimizing classification. The final cluster solution was selected based on interpretability and the percent of the study population in each cluster. Reliability and stability of the final cluster solution was tested by randomly taking a subsample (50%) of the total sample and repeating the analyses on this subsample. To check agreement, a kappa statistic was calculated between the cluster solutions of the subsample and that of the total sample.

Pearson’s chi-square tests were used to investigate the differences in cluster distribution by gender, parental education level, family SES level, and BMI status. Independent *t*-tests were used to compare physical activities, sedentary behaviors and sleep time across clusters stratified by gender and age group. General linear models were used to estimate multivariate means for food consumption and dietary pattern scores across clusters adjusted for age and energy intake. Logistic regression analysis was used to explore the odds ratios for obesity and overweight among lifestyle patterns. The models were adjusted for energy intake, sex, age, family educational level and socio-economic status (SES). Statistical tests were two-tailed with a 5% level of significance.

## 3. Results

### 3.1. Sample Characteristics

After exclusion of outliers and participants with incomplete data, 415 children and adolescents were included in the analysis. Characteristics of the sample are described in the Supplementary Materials Table S2. There was no significant difference in sociodemographic characteristics between children and adolescents. Prevalence of overweight and obesity was significantly higher in children.

### 3.2. Dietary Patterns

**Figure 1:**
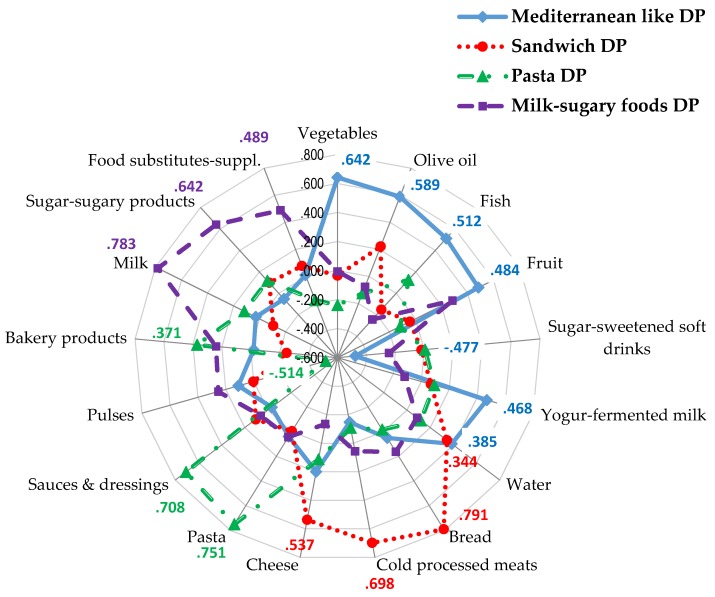
Factor loadings after varimax rotation on identified dietary patterns of food groups retained. Eigenvalues: Mediterranean like Dietary Pattern (DP) = 2.13; Sandwich DP = 1.74; Pasta DP = 1.57; Milk-sugary foods DP = 1.54. % variance explained: Mediterranean like DP: 11.37%; Sandwich DP: 10.0%; Pasta DP: 9.85%; Milk-sugary foods DP: 9.80%. Total variance explained 41.03%. Absolute values less than 0.30 are not shown.

Dietary patterns were computed for the entire sample. Bartlett’s test of sphericity and KMO = 0.601 supported the appropriateness of factor analysis. Four major factors were extracted through factor analysis using 38 food groups, which explained 41% of the variance in the model. DP1 (Mediterranean like DP) had high positive loadings on vegetables, olive oil, fish, fruits, yogurt, and fermented milk products, and water and negative loading on sugar-sweetened soft drinks. This pattern is close to the traditional Mediterranean diet. DP2 (Sandwich DP) was characterized by high positive loadings on bread, cold and processed meat products, and cheese. This pattern is closer to a “sandwich-eater” pattern. DP3 (Pasta DP) had high positive loadings on pasta, sauces, and dressings, and baked goods and high negative loadings on legumes. DP4 (Milk-sugary foods DP) showed high positive loadings on milk, sugar, and sugary foods, and food substitutes (Figure 1).

Mediterranean like DP factor scores adjusted for age and energy intake were significantly higher in girls (0.13 ± 0.07 (95% CI: −0.02–0.29) than boys (−0.07 ± 0.06 (95% CI: −0.19–0.04)).

### 3.3. Lifestyle Patterns

Based on the four identified DPs, minutes per day of vigorous and moderate physical activity, walking, biking, sedentary screen time, and sleep duration on weekdays, the two-cluster solution was found to be adequate and meaningful regarding the different patterns. Kappa statistic (κ = 0.74) suggested good agreement.

Differential characteristics of each cluster are identified by high (above 0) or low Z-scores (below 0) comparing cluster centers in Z-scores (Figure S1). Children and adolescents aggregated into cluster 1 had low scores on moderate (Z-score = −0.42) and vigorous physical activity (Z-score = −0.30), walking (Z-score = −0.30), biking (Z-score = −0.14), sleep time (Z-score = −0.07), Mediterranean like DP (Z-score = −0.11), and scored positively on sedentary screen time (Z-score = 0.02). Clustering of these behaviors (*low physical activity-poorer diet-Unhealthier lifestyle pattern*) is likely to favor a positive energy balance. Children in cluster 2 scored negatively on sedentary screen time (Z-score = –0.08) and had positive scores for sleep time (Z-score = 0.24), Mediterranean like DP (Z-score = 0.38), moderate physical activity (Z-score = 1.48) and vigorous physical (Z-score = 1.00). Clustering of these behaviors (*high physical activity, low sedentary behavior, longer sleep duration, healthier diet-Healthier lifestyle pattern*) is likely suggestive of healthier energy balance.

Characteristics of the children classified in different clusters are described in Table 1. The Unhealthier lifestyle pattern (*low physical activity-poorer diet*) included 76.9% of the sample and a significant higher proportion of girls than the Healthier lifestyle pattern. There were no significant differences regarding age group, family education, SES level or BMI status, although a higher percentage of children and adolescents included in the Unhealthier lifestyle pattern (*low physical activity-poorer diet*) were from low family SES level and were obese.

**Table 1:** Gender, age group, family educational and SES levels, and BMI status, by lifestyle pattern ^a^.

Characteristics	*Unhealthier Lifestyle* Pattern	*Healthier Lifestyle* Pattern	χ^2^
*N* (%)	319 (76.9%)	96 (23.1%)	
Gender	*N* (%)	*N* (%)	
Boys	186 (58.3%)	72 (75.0%)	8.74 *
Girls	133 (41.7%)	24 (25.0%)	
**Age group**			2.74
Children (9–12 years)	152 (47.6%)	55 (57.3%)	
Adolescents (13–17 years)	167 (52.4%)	41 (42.7%)	
**Parental educational level**			1.46
Primary or less	107 (33.5%)	28 (29.2%)	
Secondary	157 (49.2%)	54 (56.3%)	
Higher	55 (17.2%)	14 (14.6%)	
**Family SES**			2.82
Low	71 (22.3%)	14 (14.6%)	
Mid low	79 (24.8%)	28 (29.2%)	
Mid Mid high-high	169 (53.0%)	54 (56.3%)	
**BMI status**			1.31
Normal weight	202 (63.3%)	64 (66.7%)	
Overweight	89 (27.9%)	27 (28.1%)	
Obese	28 (8.8%)	5 (5.2%)	

^a^ Unhealthier lifestyle pattern: *Low physical activity-poorer diet*. Healthier lifestyle pattern: *High physical activity, low sedentary behavior, longer sleep duration, healthier diet*. Pearson’s chi-square tests were used to investigate the differences in lifestyle pattern distribution by gender, parental education level, family SES level, and BMI status; * *p* < 0.01.

Table 2 describes physical activity behaviors, sedentary screen time, sleep time on weekdays, and dietary pattern Z-scores in the final clusters in children and adolescents. Vigorous physical activity, moderate physical activity, walking time as well as Z-scores of Mediterranean like DP, were significantly higher in children and adolescents included in the healthier lifestyle pattern, but not sedentary screen time. Adolescent girls in the healthier lifestyle pattern were significantly older than those in the unhealthier lifestyle pattern.

Consumption of selected food groups and beverages by lifestyle pattern in boys and girls is described on Table 3. Consumption of vegetables, fruit, fish, yogurt, water and juices was significantly higher in boys and girls included in the healthier lifestyle pattern, as well as consumption of cheese among girls. Conversely, consumption of sugar sweetened soft drinks was higher in boys and girls in unhealthier lifestyle pattern.

Prevalence of overweight was compared between lifestyle patterns, adjusting for sociodemographic characteristics and energy intake separately in children and adolescents (Table 4). The prevalence odds ratio (PR) for overweight was not significantly different in children or adolescents allocated into different lifestyle patterns, although PR for adolescents allocated in the unhealthier lifestyle pattern was 2.00 (IC 95% 0.87–4.86) compared to those in the healthier lifestyle pattern. Although not significantly different, both in children and in adolescents the prevalence odds ratio was higher for low–mid low family SES compared to high–mid high SES level and in children, for those from lower family educational level.

**Table 2:** Physical activity behaviors, sedentary screen time, sleep time on weekdays, and dietary patterns Z-score in the final lifestyle patterns ^a^ in children and adolescents by gender.

Variables	Boys				Girls			
	Children		Adolescents		Children		Adolescents	
	Unhealthier Lifestyle Pattern	Healthier Lifestyle Pattern	Unhealthier Lifestyle Pattern	Healthier Lifestyle Pattern	Unhealthier Lifestyle Pattern	Healthier Lifestyle Pattern	Unhealthier Lifestyle Pattern	Healthier Lifestyle Pattern
	Mean (SD)	Mean (SD)	Mean (SD)	Mean (SD)	Mean (SD)	Mean (SD)	Mean (SD)	Mean (SD)
Age (year)	10.3 (1.1)	10.3 (1.1)	15.2 (1.5)	14.9 (1.5)	10.5 (1.2)	10.4 (1.2)	14.9 (1.5)	15.9 (1.0) ^†^
Sleep time week days (h/d)	8.9 (0.9)	9.1 (1.1)	8.0 (0.8)	8.4 (1.1)	9.0 (0.9)	9.1 (1.0)	7.9 (1.0)	8.3 (1.2)
Sedentary screen time (min/d)	233 (141)	214 (107)	313 (147)	294 (204)	211 (107)	245 (148)	291 (185)	259 (145)
Vigorous PA (min/d)	29 (25)	93 (50) *	30 (31)	72 (57) *	18 (30)	57 (58) *	9 (14)	90 (69) *
Moderate PA (min/d)	27 (24)	114 (51) *	18 (21)	103 (50) *	23 (25)	121 (48) *	13 (14)	98 (38) *
Walking (min/d)	37 (29)	105 (60) *	37 (33)	103 (57) *	42 (30)	90 (54) *	43 (36)	80 (53) *
Biking (min/d)	5 (11)	8 (14)	2 (5)	18 (38) *	1 (4)	9 (18)	1 (4)	1 (2)
Total PA (min/d)	111 (51)	385 (175) *	99 (53)	328 (127) *	98 (51)	385 (175) *	77 (47)	292 (89) *
Mediterranean like DP (Z-score)	−0.08 (0.86)	0.45 (0.95) ^#^	−0.28 (0.88)	0.23 (1.35) ^#^	0.13 (1.0)	0.35 (0.95) ^#^	−0.16 (0.96)	0.62 (0.47) ^#^
Sandwich DP (Z-score)	0.01 (0.94)	−0.11 (0.95)	0.24 (1.05)	0.12 (1.38)	−0.18 (0.95)	−0.26 (0.95)	−0.30 (0.80)	0.01 (0.96)
Pasta DP (Z-score)	0.08 (0.92)	0.03 (1.19)	0.09 (1.16)	0.05 (1.05)	−0.09 (0.87)	0.09 (1.19)	−0.20 (0.81)	−0.19 (0.64)
Milk-sugary foods DP (Z-score)	0.17 (0.81)	0.08 (0.80)	0.19 (1.35)	−0.17 (1.09)	−0.01 (0.76)	0.08 (0.80)	−0.24 (0.89)	−0.59 (0.55)

^a^ Unhealthier lifestyle pattern: *Low physical activity-poorer diet*. Healthier lifestyle pattern: *High physical activity*, *low sedentary behavior*, *longer sleep duration, healthier diet*. Independent *t*-tests were used to compare physical activities, sedentary behaviors and sleep time across lifestyle patterns stratified by gender and age group. General linear models were used to estimate multivariate means for dietary pattern scores across lifestyle patterns adjusted for age and energy intake; * *p* < 0.0001; ^†^ *p* < 0.05 for independent *t*-tests; ^#^ *p* < 0.01 General Linear Models adjusted for age and energy intake.

**Table 3:** Mean and median consumption of selected food groups and beverages by lifestyle pattern ^a^ in boys and girls ^b^.

Food Groups	Boys				Girls			
	Unhealthier Lifestyle Pattern		Healthier Lifestyle Pattern		Unhealthier Lifestyle Pattern		Healthier Lifestyle Pattern	
	Mean (SEM)	Median	Mean (SEM)	Median	Mean (SEM)	Median	Mean (SEM)	Median
Olive oil (ml/d)	15.4 (0.1)	15.1	17.7 (0.2) *	17.5	15.5 (0.2)	15.5	13.5 (0.5) *	13.7
Vegetables (g/d)	117 (1.0)	114	137 (1.7) *	137	128 (1.2)	126	148 (3.3) *	147
Fruit (g/d)	88.7 (2.1)	88.3	122 (2.8) *	121	106 (2.0)	108	147 (6.1) *	147
Pulses (g/d)	12.9 (0.1)	12.8	13.3 (0.2)	13.3	13.2 (0.1)	13.3	13.4 (0.2)	13.5
Fish (g/d)	37.8 (0.3)	37.8	55.2 (0.5) *	55.7	41.9 (0.2)	42.0	80.4 (0.7) *	80.7
Bread (g/d)	95.5 (1.8)	90.9	96.7 (3.1)	97.2	80.8 (1.6)	78.8	79 (4.5)	79.6
Pasta (g/d)	24.7 (0.8)	23.9	23.8 (0.6)	23.9	19.1 (0.2)	19.3	15.5 (0.6)	15.8
Bakery products (g/d)	48.3 (1.3)	46.4	46.1 (2.3)	46.7	41.1 (0.8)	40.4	48.4 (2.4)	48.8
Sugar and sugary products (g/d)	23.8 (0.3)	23.5	20.8 (0.6) *	20.7	24.0 (0.6)	23.4	24.1 (1.8)	24.3
Milk (g/d)	274 (2.8)	267	247 (4.8) *	248	208 (2.6)	211	209 (7.8)	215
Cheese (g/d)	16.0 (0.4)	14.9	17.2 (0.6)	17.1	15.0 (0.4)	14.5	21.9 (0.9) *	21.8
Yoghurt and fermented milk (g/d)	51.8 (1.3)	53.2	73.3 (2.2) *	74.9	50.2 (1.6)	51.8	69.1 (4.8) *	72.2
Meats (g/d)	105 (1.0)	103	117 (1.5) *	116	88.6 (1.2)	88.7	79.4 (2.7) *	79.5
Cold and processed meats (g/d)	59.4 (0.9)	57.3	51.4 (1.6)	51.6	48.7 (0.9)	49.2	50.6 (2.9)	51.9
Water (g/d)	582 (5.2)	578	627 (9.3) *	625	526 (0.8)	527	629 (2.3) *	630
Sugared soft drinks (g/d)	143 (3.8)	138	98.1 (5.2) *	91.4	95.6 (2.9)	95.1	66.8 (6.8) *	50.3
Juices (g/d)	115 (1.9)	115	126 (3.5) *	125	114 (1.9)	111	159 (5.3) *	160
Sauces and dressings (g/d)	15.4 (0.2)	15.2	15.8 (0.4)	15.8	14. (0.2)	14.4	15.4 (0.7)	15.7

^a^ Unhealthier lifestyle pattern: *Low physical activity-poorer diet*. Healthier lifestyle pattern: *High physical activity*, *low sedentary behavior*, *longer sleep duration*, *healthier diet*. ^b^ General linear models were used to estimate multivariate means for food consumption and dietary pattern scores across lifestyle patterns adjusted for age and energy intake; * *p* < 0.05.

**Table 4:** Prevalence ratio (PR) of overweight/obesity in children and adolescents, according to gender, age, family level of education, socioeconomic level and lifestyle pattern ^a^ in children and in adolescents.

Variables	Children				Adolescents			
	PR	95% C.I.PR		*p*	PR	95% C.I.PR		*p*
		Lower	Upper			Lower	Upper	
**Gender**								
Girls	1				1			
Boys	1.26	0.70	2.25	NS	1.79	0.85	3.76	NS
Age	0.92	0.72	1.18	NS	0.78	0.62	0.97	0.026
Family Level of education				NS				NS
High	1				1			
Secondary	1.00	0.46	2.19	NS	0.67	0.26	1.17	NS
Primary	1.15	0.49	2.69	NS	0.52	0.19	1.43	NS
SES				NS				NS
Mid-high-High	1				1			
Mid	0.88	0.42	1.87	NS	0.66	0.27	1.59	NS
Mid-low-Low	1.27	0.65	2.50	NS	1.88	0.87	4.06	NS
**Lifestyle pattern**								
Healthier lifestyle patter					1			
Unhealthier lifestyle pattern	1.07	0.55	2.04	NS	2.00	0.82	4.86	NS

Note: Binary logistic regression models adjusted for age, sex, family SES, family educational level and energy intake. ^a^ Unhealthier lifestyle pattern: *Low physical activity-poorer diet*. Healthier lifestyle pattern: *High physical activity, low sedentary behavior, longer sleep duration, healthier diet*.

## 4. Discussion

This study showed that dietary patterns, physical activity behaviors, sedentary behaviors and sleep time on weekdays in Spanish children and adolescents cluster in two different groups. A *low physical activity-poorer diet* lifestyle pattern (Unhealthier lifestyle pattern), which included a higher proportion of girls, and a *high physical activity, low sedentary behavior, longer sleep duration, healthier diet* lifestyle pattern (Healthier lifestyle pattern). Although increased risk of being overweight was not significant, the ORs for the unhealthier lifestyle pattern (*low physical activity-poorer diet)* were >1 in children and in adolescents.

Analysis of dietary patterns has been increasingly used to consider total food intake and the potentially synergistic effects of foods and nutrients [10,18]. Factor analysis and cluster analysis are procedures commonly used for that purpose. In this study factor analysis was used to identify dietary patterns that were later used in cluster analysis. This approach has been used by other authors [37]. Several studies have identified a healthier or traditional DP in children and adolescents, with higher scores on fruits and vegetables and lower scores in energy-dense foods [7,11,12,13,14,15,16,17,38,39], such as the Mediterranean like DP identified in this study. A DP high in sandwiches or packed lunches, similar to that identified in this analysis, has also been described in different studies [7,10,40].

The results of cluster analysis in this study are consistent with findings by other authors reporting a healthy energy balance-related behavior pattern that combines a healthier diet with high levels of physical activity and low levels of sedentary behavior, among children and adolescents in different countries [13,22,32,40,41,42,43,44,45]. Several studies also report the clustering of a combination of sedentary lifestyle with healthy diet [44,45]. A systematic review on the clustering of diet, physical activity, and sedentary behavior among children and adolescents aged 9–21 years, except one study of children aged 5–12 years, found that most children and adolescents in those studies fell into a mixed category of one or more healthy behaviors together with one or more unhealthy behaviors [18]. Te Velde *et al.* also identified a cluster with low physical activity and low sedentary behaviors in 10- to 12-year-old European children, with a higher proportion of girls [46].

In our study, sleep time scored positively in the healthier lifestyle pattern. A long sleep duration-physically inactive lifestyle pattern was identified among European children aged 10–12 years [32]. The same study also identified a short sleep duration-physically inactive lifestyle pattern. Sleep quality is an issue, which deserves further research in addition to sleep duration.

The healthier lifestyle pattern included 23% of children and adolescents in our study and a significantly different gender distribution; this lifestyle pattern characterized by high physical activity and healthier diet had a lower proportion of girls (25%). Several studies have reported that boys score higher than girls on less healthy dietary patterns or poorer diet-sedentary behaviors, such as observations from the KOALA Birth Cohort Study in The Netherlands [23] and the Young Finns Study [11]. Boys have also been reported to cluster into a high physical activity-healthy diet pattern or a high physical activity-high sedentary pattern [44]. In our analysis, Mediterranean like DP factor scores adjusted for age and energy intake were significantly higher in girls in line with findings that report girls to be more likely within a healthy or traditional dietary pattern [47].

Differences between boys and girls in sedentary and physical activity behaviors have been reported by other authors [46,48]. Gender difference in physical activity seems consistent in several studies, girls being less physically active particularly in adolescents [20]. This was also reported in the PERSEO project (Pilot Reference School Program for Health and Physical Actitvity against Obesity)—a school based intervention aimed to foster healthier eating and physical activity behaviors in Primary School children—among Spanish school children [49]. A consistent finding in the review by Leech *et al.* [18] was a higher proportion of boys in the high physical activity lifestyle patterns and more girls in those with low physical activity, as observed in our analysis.

Contradictory results have been reported regarding sedentary behavior [50]. Jago *et al.* [51] suggested that girls engage in sedentary activities that are often not assessed, such as personal care and social interactions.

In this study, the unhealthier lifestyle pattern (*low physical activity-poorer diet*) included a higher proportion of families with a low SES level (22.3%). Leech *et al.* [18] reported in their systematic review a higher proportion of children or adolescents from a low SES in the lifestyle patterns defined by low levels of physical activity. Some evidence has also been found suggesting that children from low SES tend to be in lifestyle patterns defined by high levels of sedentary behaviors. However, some studies have found no association between cluster grouping and SES [44].

An inverse association between parental education and sedentary behavior has been observed in other studies. In their systematic review, Leech *et al.* found that lifestyle patterns characterized by high physical activity or participation in sports were significantly associated with higher levels of parental education, adolescent education, and family income [18]. High sedentary behavior lifestyle patterns were associated with low parental education and low family income levels [17,20]. We found no significant association with family educational level, although a higher proportion of children from lower education families were included in the low physical activity lifestyle pattern.

In this study, increased risk of being overweight was not significant across lifestyle patterns in children or adolescents. Systematic reviews on this issue have concluded that the evidence is inconsistent with respect to a cumulative effect of these behaviors on obesity outcomes; some studies have found a higher prevalence of overweight and obesity in unhealthy lifestyle patterns, whereas others have found no association at all. In the review by Leech *et al.*, five studies, including two longitudinal studies, showed evidence of a possible synergistic effect of multiple unhealthy behaviors on overweight or obesity, two studies found an unexpected inverse association with an unhealthy lifestyle pattern, and seven studies found no association [18]. In those studies that found a possible synergistic effect, lifestyle patterns characterized by either low physical activity or high sedentary behavior were positively associated with overweight [52]. A pattern combining inactivity and sedentary behavior with television viewing during meals was related to increased cross-sectional odds of overweight in boys in the Pro Children study among 10–12 year-old European school children [46]. This inconsistency can likely be attributed to the cross-sectional design of many of these studies, but it also reflects the complexity of energy balance-related behavior, sociodemographic correlates, other determinant factors, and their relationships with obesity. To date, most studies investigating dietary patterns have focused on food consumption but do not consider eating routines, how, when, or where people eat. Several studies have found that determinants of energy balance-related behaviors also cluster, such as parenting practices [10,23].

Strengths of this study include the careful design, protocol, and methodology used in the ANIBES study, conducted among a random representative sample of the Spanish population aged 9–75 years. Food consumption assessment was made using digital tablets and included a thorough quality control process. The use of factor analysis allowed us to identify dietary patterns without any *a priori* defined criteria and their inclusion in cluster analysis together with physical activity, sedentary behaviors, and sleep time provided a broader perspective.

One limitation of this study is the cross-sectional design, which provides evidence for association but not causal relationships, and residual confounding by unobserved and unmeasured factors is likely. Measures of food consumption and physical activity relied on self-reports and were possibly biased, although a careful multistep quality control procedure was implemented to minimize bias. However, misreporting can influence the potential association with overweight. Information on physical activity and sedentary behaviors used in this analysis was self-reported as well. Accelerometers were used to collect data in a subsample and showed modest significant correlations with vigorous physical activity (*r* = 0.26), in line with those observed in validation studies of a modified version of IPAQ for children and adolescents [28]. The validity correlation coefficients from the majority of existing PAQs has been considered poor to moderate and it has been suggested that most PAQs may be valid for ranking individuals’ behavior rather than quantifying physical activity. In this analysis we used Z-scores of time spent on physical activity and sedentary behaviors, capturing ranking rather than absolute magnitude. Time spent in physical activities was used to describe cluster characteristics.

In addition, we used factor analysis to identify DPs based on food consumption information collected by three-day food records and one 24-h recall, considering food groups to further collapse dietary intake data and avoid missing data from non-consumers of episodically consumed foods. This procedure has been used by other authors [37], although Food Frequency Questionnaires (FFQ) data has often been used as input source to identify dietary patterns. Similar DPs have been identified in adolescents using food records or FFQ as input data [42]. It has been argued whether food consumption data should be energy adjusted for such analysis. Smith *et al.* [53] did not find obvious differences between the patterns derived by principal component analysis on three-day diet diary data in children using percentage energy or adjusting weight for total energy intake compared to those derived using gram weights.

Finally, both factor analysis and cluster analysis are procedures commonly used to identify DPs and analyze clustering of lifestyles. These procedures rely on several subjective decisions that may influence outcomes regarding the number and type of patterns and clusters identified.

## 5. Conclusions

Four dietary patterns were identified, one closer to the traditional Mediterranean diet. Cluster analysis classified Spanish children and adolescents in two different groups. A *low physical activity-poorer diet* lifestyle pattern (Unhealthier lifestyle pattern), and *a high physical activity, low sedentary behavior, longer sleep duration, healthier diet* lifestyle pattern (Healthier lifestyle pattern). The prevalence odds ratio for overweight and obesity was not significantly different in children or adolescents allocated into the different clusters, although the ORs for the *low physical activity-poorer diet* lifestyle pattern were >1 in children and in adolescents. Prospective research using larger samples is needed to further examine how lifestyle patterns of energy balance-related behaviors track over time and influence the development of overweight and obesity. These behavior patterns are helpful to identify specific issues and suggest potential intervention strategies.

## Supplementary Materials

Supplementary materials can be accessed at: http://www.mdpi.com/2072-6643/8/1/0011/s1.
