# Supplementary material for: Clustering of Dietary Patterns, Lifestyles, and Overweight among Spanish Children and Adolescents in the ANIBES Study
Source: Nutrients. 2015 Dec 28;8(1):11. doi: 10.3390/nu8010011 (PMC4728625; doi:10.3390/nu8010011)
Supplement: Supplementary file 1 [file nutrients-08-00011-s001.docx]

Supplementary Materials: Clustering of Dietary Patterns, Lifestyles, and Overweight among Spanish Children and Adolescents in the ANIBES Study

Carmen Pérez-Rodrigo, Ángel Gil, Marcela González-Gross, Rosa M. Ortega,
Lluis Serra-Majem, Gregorio Varela-Moreiras and Javier Aranceta-Bartrina

**Table S1.** Food groups and subgroups in the ANIBES Study.

| **Food Groups and Subgroups** | **Description** |
| --- | --- |
| **Grains** |  |
| Grains and flours |  |
| Bread | All types of bread, including whole grain |
| Breakfast cereals and cereal bars |  |
| Pasta |  |
| Bakery and pastry | Biscuits, sweet breads and cakes |
| **Vegetables** | All vegetables, including root vegetables and potatoes |
| **Fruits** | All fresh, canned, and dried fruits, including nuts and seeds |
| **Oils and fats** |  |
| Olive oil | Olive oil and virgin olive oil |
| Other oils |  |
| Butter, margarine and shortening |  |
| **Milk and dairy products** |  |
| Milks | All types of milk |
| Cheeses | All types of cheese |
| Yogurt and fermented milk | All yoghurt and fermented milk products |
| Other dairy products |  |
| **Fish and Shellfish** |  |
| **Meat and meat products** |  |
| Meat | All meat and meat products |
| Sausages and other meat products | Sausages, cold and processed meats |
| Viscera and spoils |  |
| **Eggs** |  |
| **Pulses** | Green and dried pulses |
| **Sugars and sweets** |  |
| Sugar |  |
| Chocolates |  |
| Jams and other | Jam, marmalade |
| Other sweets | Sweets, confectionery, turron |
| **Appetizers** |  |
| **Ready-to-eat-meals** |  |
| **Sauces and condiments** | Sauces, dressings, condiments, and spices |
| **Non-alcoholic beverage** |  |
| Water |  |
| Coffee and infusions |  |
| Sugar soft drinks | Any flavor sugared soft drinks and sodas |
| Non-sweetened soft drinks |  |
| Sports Drinks |  |
| Energy drinks |  |
| Juices and nectars | Juices and nectars, any flavor |
| Other drinks |  |
| **Alcoholic beverages** |  |
| Low alcohol content beverages |  |
| High alcohol content beverages |  |
| **Supplements and meal replacement** |  |

**Table S2.** Characteristics of sample included in the analysis.

| **Characteristics** | **Total (*n* = 415)** | **Children (*n* = 207)** | **Adolescents (*n* = 208)** |
| --- | --- | --- | --- |
|  | **Mean (SD)** | **Mean (SD)** | **Mean (SD)** |
| Age | 12.7 (2.7) | 10.4 (1.1) | 15.1 (1.5) |
| Gender | *N* (%) | *N* (%) | *N* (%) |
| Boys | 258 (62.2) | 123 (59.5) | 135 (64.9) |
| Girls | 157 (37.8) | 84 (40.6) | 73 (35.1) |
| Habitat | | | |
| 20,000–30,000 inhabitants | 156 (37.6) | 77 (37.2) | 79 (38.0) |
| >30,000–200,000 inhabitants | 157 (37.8) | 79 (38.2) | 78 (37.5) |
| >200,000 inhabitants | 102 (24.6) | 51 (24.6) | 51 (24.5) |
| Parental educational level | | | |
| Primary or less | 135 (32.5) | 65 (31.4) | 70 (33.7) |
| Secondary | 211 (50.8) | 104 (50.2) | 107 (51.4) |
| Higher | 69 (16.6) | 38 (18.4) | 31 (14.9) |
| Family SES | | | |
| Low | 85 (20.5) | 41 (19.8) | 44 (21.2) |
| Mid-low | 107 (25.8) | 55 (26.6) | 52 (25.0) |
| Mid | 201 (48.4) | 100 (48.3) | 101 (48.6) |
| Mid high-high | 22 (5.3) | 11 (5.3) | 11 (5.3) |
| BMI status * | | | |
| Normal weight | 266 (64.1) | 116 (56.0) | 150 (72.1) |
| Overweight | 116 (28.0) | 70 (33.8) | 46 (22.1) |
| Obese | 33 (8.0) | 21 (10.1) | 12 (5.8) |

* Pearson’s Chi-Squared test = 11.764 (*p* = 0.003).

**Figure S1.** Standard deviation scores of cluster centers on dietary patterns identified, minutes per day of vigorous and moderate physical activity, walking, and biking, as well as sleep duration on weekdays in Spanish children and adolescents.
